# Development of a physiological model of human middle ear epithelium

**DOI:** 10.1002/lio2.661

**Published:** 2021-09-18

**Authors:** Michael William Mather, Bernard Verdon, Rachel Anne Botting, Justin Engelbert, Livia Delpiano, Xin Xu, Catherine Hatton, Tracey Davey, Steven Lisgo, Philip Yates, Nicholas Dawe, Colin D. Bingle, Muzlifah Haniffa, Jason Powell, Chris Ward

**Affiliations:** ^1^ Faculty of Medical Sciences Biosciences Institute, Newcastle University Newcastle‐upon‐Tyne UK; ^2^ Department of Otolaryngology Freeman Hospital Newcastle‐upon‐Tyne UK; ^3^ Newcastle Biobank, Faculty of Medical Sciences Newcastle University Newcastle‐upon‐Tyne UK; ^4^ Faculty of Medical Sciences Translational and Clinical Research Institute, Newcastle University Newcastle‐upon‐Tyne UK; ^5^ Electron Microscopy Research Services, Faculty of Medical Sciences Newcastle University Newcastle‐upon‐Tyne UK; ^6^ Department of Infection, Immunity and Cardiovascular Disease The Medical School Sheffield UK

**Keywords:** biological models, otitis media, otorhinolaryngologic diseases, respiratory mucosa, SARS‐CoV‐2

## Abstract

**Introduction:**

Otitis media is an umbrella term for middle ear inflammation; ranging from acute infection to chronic mucosal disease. It is a leading cause of antimicrobial therapy prescriptions and surgery in children. Despite this, treatments have changed little in over 50 years. Research has been limited by the lack of physiological models of middle ear epithelium.

**Methods:**

We develop a novel human middle ear epithelial culture using an air‐liquid interface (ALI) system; akin to the healthy ventilated middle ear in vivo. We validate this using immunohistochemistry, immunofluorescence, scanning and transmission electron microscopy, and membrane conductance studies. We also utilize this model to perform a pilot challenge of middle ear epithelial cells with SARS‐CoV‐2.

**Results:**

We demonstrate that human middle ear epithelial cells cultured at an ALI undergo mucociliary differentiation to produce diverse epithelial subtypes including basal (p63+), goblet (MUC5AC+, MUC5B+), and ciliated (FOXJ1+) cells. Mature ciliagenesis is visualized and tight junction formation is shown with electron microscopy, and confirmed by membrane conductance. Together, these demonstrate this model reflects the complex epithelial cell types which exist in vivo. Following SARS‐CoV‐2 challenge, human middle ear epithelium shows positive viral uptake, as measured by polymerase chain reaction and immunohistochemistry.

**Conclusion:**

We describe a novel physiological system to study the human middle ear. This can be utilized for translational research into middle ear diseases. We also demonstrate, for the first time under controlled conditions, that human middle ear epithelium is susceptible to SARS‐CoV‐2 infection, which has important clinical implications for safe otological surgery.

**Level of Evidence:**

NA.

## 1 INTRODUCTION

Inflammation of the middle ear, known generally as otitis media (OM), represents a spectrum of disease. This can be initiated by an acute infection in acute otitis media and may lead to a persistent accumulation of fluid in the middle ear cavity in otitis media with effusion or to chronic suppurative otitis media when associated with a perforated tympanic membrane.

The burden of OM is substantial. It is one of the largest causes of infections and surgery in childhood^1^ and OM‐related hearing loss affects 30 per 10 000 people.^2^ Despite the substantial morbidity of OM, few in vitro models of middle ear epithelium exist. Air‐liquid interface (ALI) cultures are well established for the generation of physiological models of airway epithelium from much of the respiratory tract, but application of this technique to the human middle ear is lacking. A comprehensive cellular model of middle ear epithelium which recapitulates key features of differentiated cell types will facilitate translational research into OM and related middle ear diseases.

Viral infection is a common cause of OM. Little is known, however, about the effect of SARS‐CoV‐2 on middle ear epithelium and whether this can induce OM akin to other viruses. We aim to perform a pilot exploration, utilizing this physiological human model of middle ear epithelium, to assess middle ear susceptibility to SARS‐CoV‐2 infection.

## 2 METHODS

### 2.1 Tissue acquisition

Human fetal middle ear epithelium (n = 1; 14 post conception weeks) was obtained from the MRC/Wellcome Trust‐funded Human Developmental Biology Resource (HDBR; http://www.hdbr.org) with written consent and approval from the Newcastle and North Tyneside NHS Health Authority Joint Ethics Committee (08/H0906/21 + 5). HDBR is regulated by the UK Human Tissue Authority (HTA; www.hta.gov.uk) and operates in accordance with the relevant HTA Codes of Practice to provide human fetal tissue from 3 to 20 post conception weeks for biomedical research. Quantitative fluorescent polymerase chain reaction (PCR) was used to exclude common aneuploidies. The temporal bone was micro‐dissected to identify the middle ear cavity and middle ear mucosal tissue was extracted.

### 2.2 Air‐liquid interface cultures

The tissue was dissociated into a cell suspension and passed through a 100‐μm cell strainer (Greiner Bio‐One, Kremsmünster, Austria) and centrifuged. Cells were seeded onto a collagen‐coated flask with PneumaCult‐Ex Plus (Stemcell Technologies) and cultured in a humidified atmosphere containing 5% CO_2_ at 37°C. Cells were expanded over 7 to 10 days with media changes every 48 to 72 hours.

Upon reaching, 70% confluence cells were passaged onto Costar transwell membranes (Sigma Aldrich, St Louis, Missouri), with 0.4 μm pore size, pre‐coated with collagen. Cells underwent expansion in the transwell system using PneumaCult‐Ex Plus (Stemcell Technologies) for 4 days with media supplied to both the apical and basolateral compartments. On day 4, the media was removed from the apical compartment and the cells were exposed to air on their apical surface only. After 4 weeks, cultures underwent subsequent validation experiments.

### 2.3 Immunohistochemistry

Transwell membranes were fixed in 4% paraformaldehyde. The specimen was dehydrated through graded ethanol baths, infiltrated with paraffin wax, and embedded into paraffin blocks at 60°C. The tissue blocks were then sectioned at 5‐μm thickness using an RM2235 Microtome (Leica). The Ventana Discovery Autostainer (Ventana, Tucson, Arizona) was used for immunohistochemistry (IHC) following the manufacturer's instructions.

### 2.4 Immunofluorescence

The transwell membranes were washed and fixed in 4% paraformaldehyde, permeabilized with 0.3% Triton and blocked with 5% bovine serum albumin and incubated with the primary and secondary antibodies (Supporting Information S1) as previously described.^3^ Transwell scaffolds were then fixed to glass slides and imaged on an Axioimager without immersion (Zeiss, Oberkochen, Germany).

### 2.5 Transmission electron microscopy

Membrane‐bound epithelial cells were fixed, dehydrated, and impregnated with increasing concentrations of epoxy resin. They were then polymerized at and sections were cut on an ultramicrotome (70 nm). Sections were stained with 1% uranyl acetate and 2% lead citrate on an EM AC20 automatic staining machine (Leica) before being viewed on a Hitachi HT7800 transmission electron microscope as described previously.^4^

### 2.6 Scanning electron microscopy

Membrane‐bound epithelial cells were fixed in 2.5% glutaraldehyde, dehydrated in graded ethanol, and placed in a Baltec CPD 030 critical point dryer (Leica), gold coated and observed under a Tescan VEGA LMU SEM microscope (Tescan, Cambridge, UK) as described previously.^4^

### 2.7 Cell membrane barrier function and ion channel characterization

Epithelial integrity and ion transport was evaluated using short‐circuit current measurements using selective ion channel inhibitors in an Ussing chamber (VCC MC 8 model, Physiologic Instruments, California). The membrane‐bound epithelial cells were inserted into the EasyMount Ussing chamber system and bathed in bilateral modified bicarbonate Kreb's solution, continuously gassed with 95% O_2_/5% CO_2_ maintained at 37°C. Cells were voltage‐clamped to 0 mV and left to equilibrate before adding the inhibitors and agonists, which were added in the following order: amiloride (10 μM apical; Sigma‐Aldrich), forskolin (10 μM, bilateral; Tocris Bioscience, Bristol, UK), CFTRinh172 (20 μM, apical; Tocris Bioscience). The transepithelial short‐circuit current (Isc) and transepithelial electrical resistance were measured using Ag and Ag/AgCl electrodes with 3 M KCl agar bridges and recorded using the Acquire & Analyze software Revision II (Physiologic Instruments). The assay was simultaneously run with duplicate samples.

### 2.8 SARS‐CoV‐2 Infection

A clinical isolate of SARS‐CoV‐2 (England/2/2020) was obtained from Public Health England. The initial viral stock was propagated in Vero E6 cells as previously described.^5^ Infections were performed in a containment level 3 facility. 2 × 10^5^ pfu of SARS‐CoV‐2 was added to the apical side of two wells of middle ear epithelial cells differentiated at an ALI and one well of confluent Vero E6 cells (passage 10). Two wells of middle ear epithelium and one well of Vero E6 cells were left untreated (mock control).

All samples were incubated (37°C/5% CO_2_) for 2 hours. Subsequently, the samples were washed and fresh media added. The cells were returned to the incubator until cell collection.

### 2.9 Quantitative Reverse Transcription (RT) PCR

Mock and SARS‐CoV‐2 infected cells were lysed with Trizol reagent (Invitrogen) for RNA extraction, with resultant RNA being reverse transcribed with Superscript III (ThermoFisher Scientific) according to manufacturer's instructions. cDNA templates underwent qPCR as previously described.^6^

Two PCR probes against the SARS‐CoV‐2 nucleocapsid (N) genes (N1/N2) were tested using the 2019‐nCoV RUO kit (Integrated DNA Technologies, Coralvillle, Iowa) in accordance with manufacturer's instruction. The qPCR was then run using an AriaMx real‐time PCR system (Agilent Technologies, California). Expression was determined by ddCT where the nucleocapsid expression was normalized to the endogenous housekeeping gene, RNAseP, and duplicate samples averaged.

Tissue preparation and IHC for anti‐SARS‐CoV‐2 was performed as outlined in the earlier methods using the anti‐SARS‐CoV‐2 spike protein antibody (Sino‐Biological, Beijing, China; 40 589‐T62).

## 3 RESULTS

### 3.1 Air‐liquid interface cultures

On the first and only attempt at running these cultures middle ear epithelial cells were observed to form discrete islands in the days following initiation of submerged culture which gradually connected one another from approximately day 7. A typical epithelial “cobblestone” morphology was witnessed as cells approached confluence at approximately 14 days.

**FIGURE 1:**
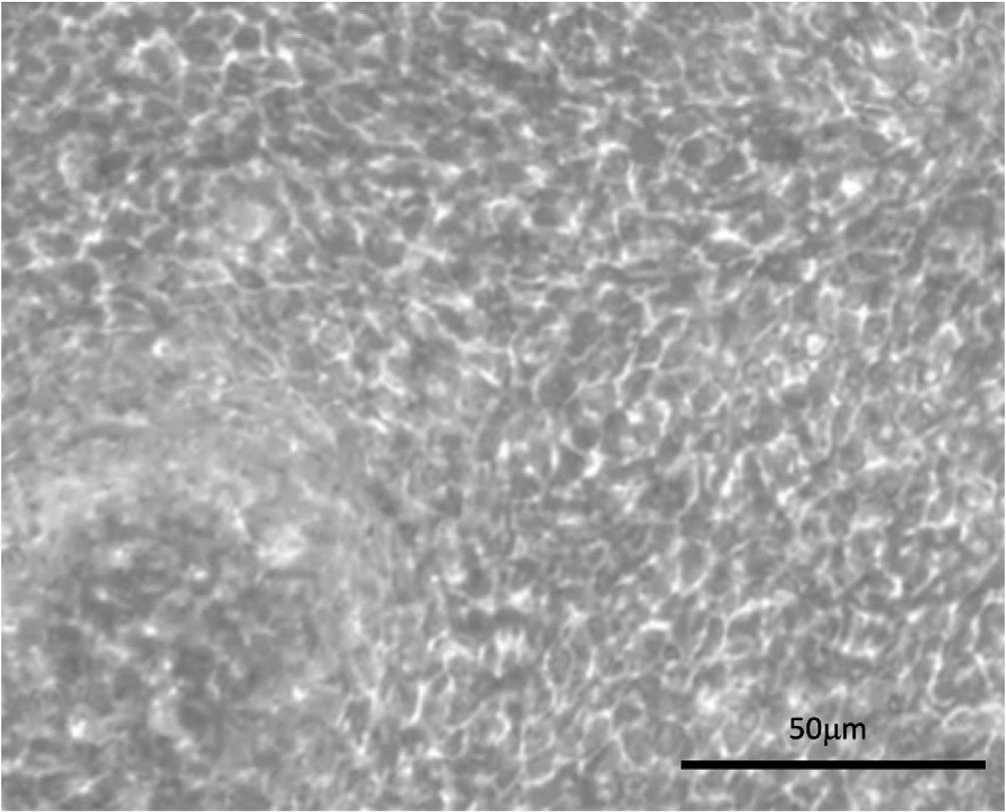
Dense distribution of epithelial cells seen on light microscopy (×20 magnification). Scale bar 50 μm

Once cultured at an ALI cells underwent mucociliary differentiation over a period of 4 weeks. After this time, they were used for cell characterization and further experiments. Macroscopically, the production of airway surface liquid was evident as a slim meniscus covering the transwell. Conventional light microscopy revealed a dense population of epithelial cells (Figure 1) with isolated areas of motile cilia (Supporting Information S2).

### 3.2 Immunohistochemistry

**FIGURE 2:**
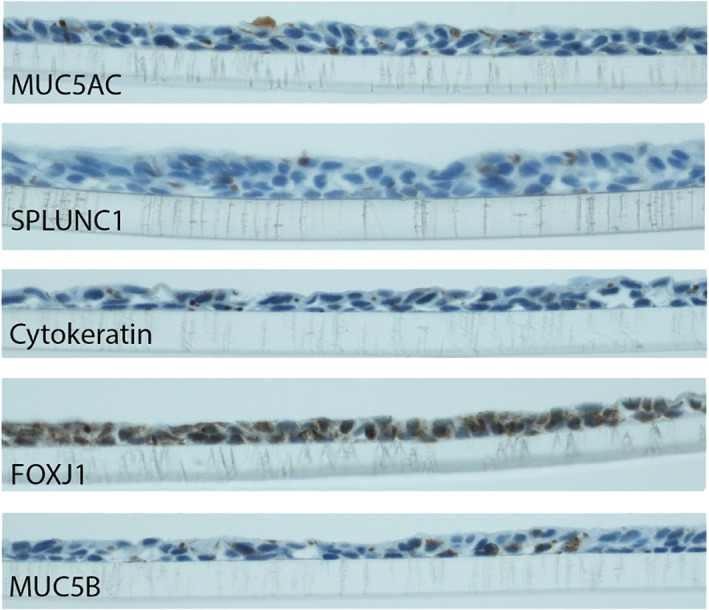
Immunohistochemistry for (top to bottom) MUC5AC, SPLUNC1, cytokeratin, FOXJ1, and MUC5B

IHC revealed strongly positive staining for FOXJ1, moderate positivity in MUC5AC and MUC5B, and mildly positive staining with SPLUNC1 and cytokeratin (Figure 2).

### 3.3 Immunofluorescence

**FIGURE 3:**
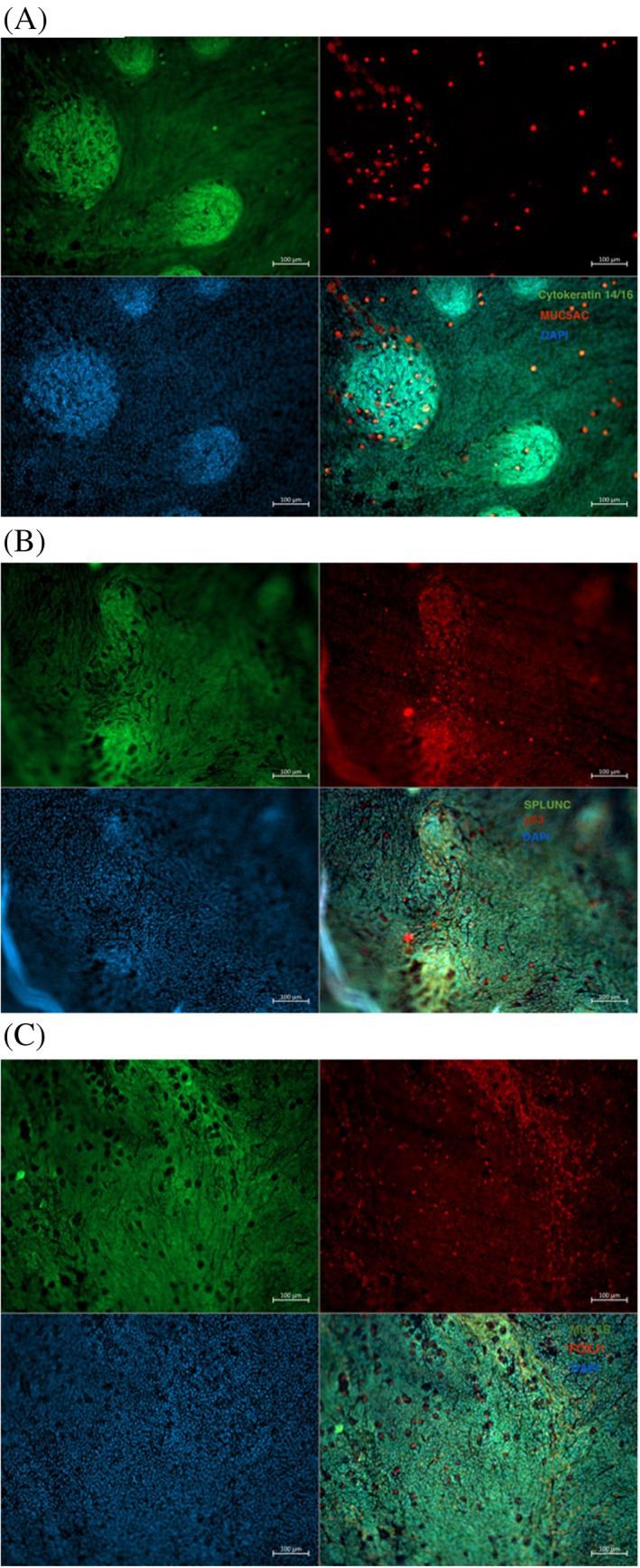
(A) Immunofluorescence with cytokeratin 14/16 [green] and MUC5AC [red] (upper left; and upper right), DAPI [blue] as a nuclear counterstain (bottom left), and the merged staining (bottom right). Scale bar 100 μm. (B) Immunofluorescence with SPLUNC [green] and p63 [red] (upper left and upper right), DAPI [blue] as a nuclear counterstain (bottom left), and the merged staining (bottom right). Scale bar 100 μm. (C) Immunofluorescence with MUC5B [green] and FOXJ1 [red] (upper left and upper right), DAPI [blue] as a nuclear counterstain (bottom left), and the merged staining (bottom right). Scale bar 100 μm

Immunofluorescence (Figure 3) revealed scattered positive staining with cytokeratin 14/16. There was strong positive staining with MUC5AC, p63, and FOXJ1. Little positivity was observed with SPLUNC and MUC5B.

### 3.4 Electron microscopy

**FIGURE 4:**
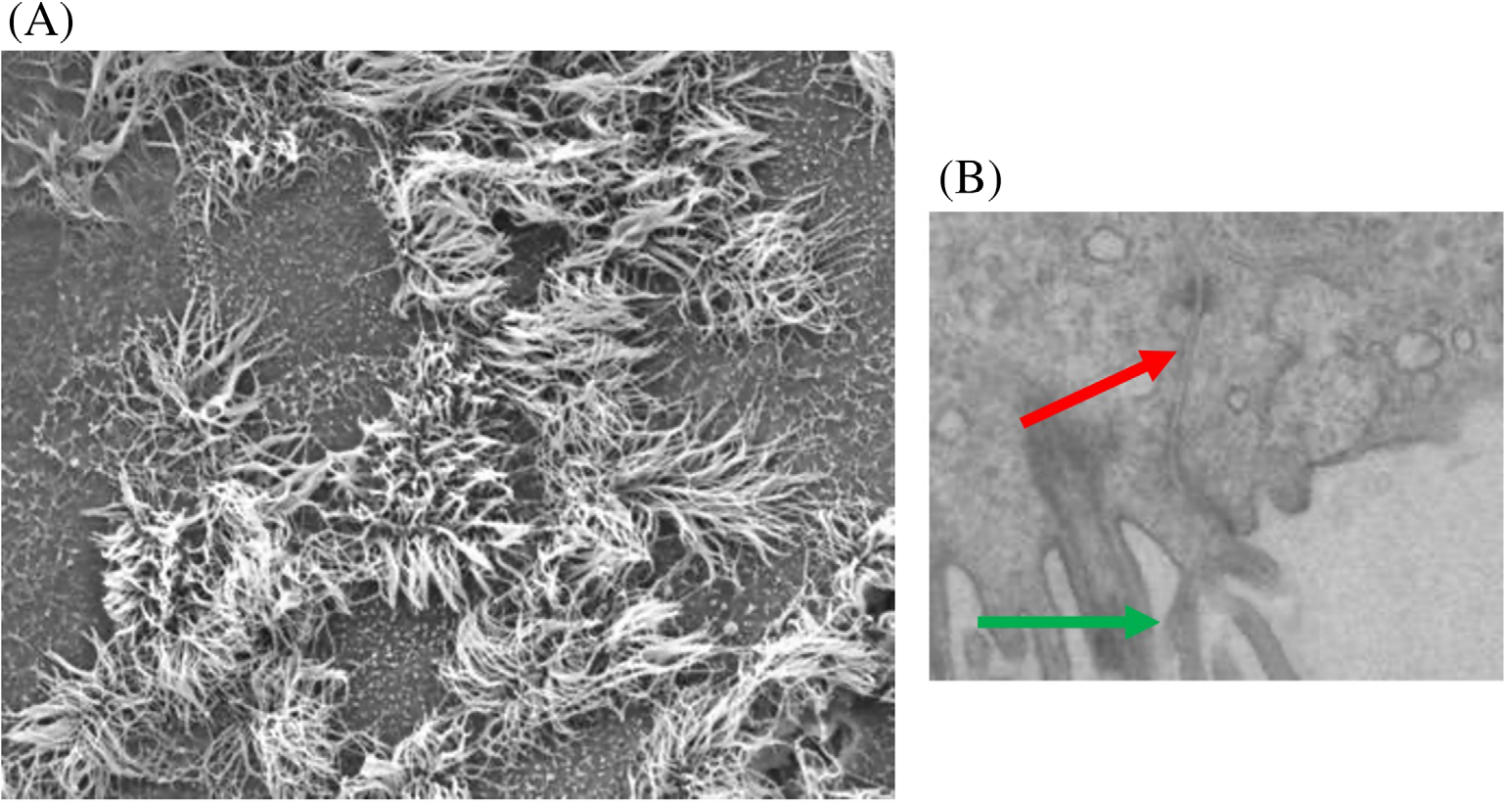
(A) Scanning electron microscopy (SEM) demonstrating presence of cilia. (B) Transmission electron microscopy (TEM) demonstrating appearances supportive of the formation of tight junctions (red arrow) and cilia (green arrow)

Scanning electron microscopy (SEM) reveals a surface with moderately dense cilia coverage (Figure 4). Transmission electron microscopy (TEM; Figure 4B) similarly demonstrates cilia (green arrow) and also the presence of tight junctions (red arrow).

### 3.5 Ussing chamber

**FIGURE 5:**
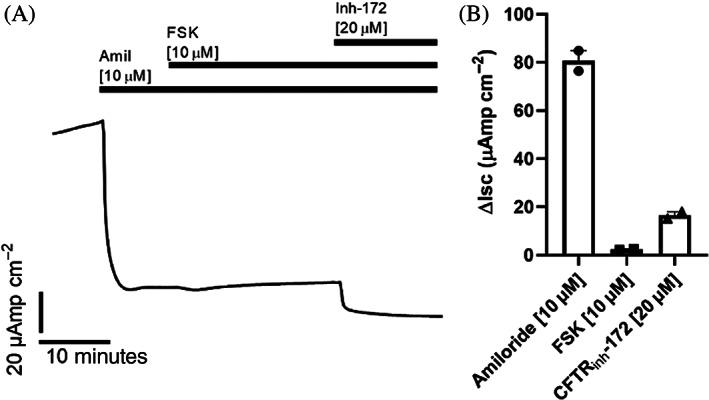
(A) Human middle ear epithelia show ENaC and CFTR activity. Mean short circuit current (Isc) measurements over time from two middle ear epithelial transwells run in duplicate. ENaC activity was measured as a change in Isc (Δisc) induced by amiloride (Amil, 10 μM, apical). CFTR activity was measured as change in Isc (ΔIsc) induced by forskolin (FSK, 10 μM, bilateral) and CFTRinh172 (Inh‐172, 20 μM, apical). (B) Absolute mean lsc with the addition of Amil, FSK, and Inh‐172

Tight epithelial junctions were confirmed by resistance measurements on Ussing chamber experiments. Selective ion channel inhibitors (Table 1) were sequentially added to assess individual ion channels; revealing an abundance of predominantly ENaC and CFTR transporters (Figure 5). A mean resting transepithelial electrical resistance (TEER) of 231.0 Ω cm^2^ was recorded, consistent with the development of epithelial barrier function.

**TABLE 1:** Short circuit current by ion channel inhibitors

	Short circuit current (Isc)/μAmp cm^−2^		
	Transwell 1	Transwell 2	Mean
Amil	−76.4	−84.9	−80.7
FSK	2.6	2.2	2.4
Inhib‐172	−15.2	−18.0	−16.6

Abbreviations: Amil, amiloride; FSK, forskolin; Inh‐172, inhibitor of cystic fibrosis transmembrane conductance regulator (CFTR).

### 3.6 SARS‐CoV‐2 infection

**FIGURE 6:**
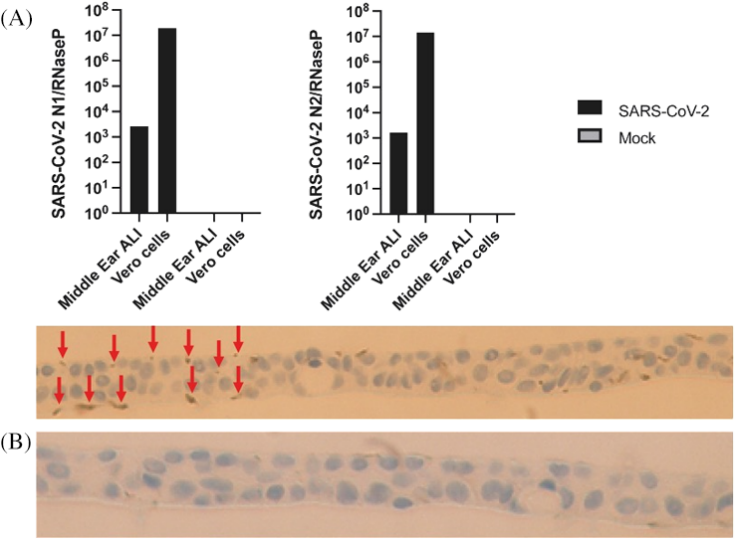
(A) In vitro SARS‐CoV‐2 infection of middle ear epithelium. RT‐PCR for SARS‐CoV‐2 N1 (left panel) and N2 (right panel) expression in SARS‐COV‐2 (black) and mock (grey) infected middle ear and vero cells and mock infected cells 24 hours post infection. (B) immunohistochemistry (IHC) of membrane‐bound middle ear epithelium with anti‐SARS‐CoV‐2 antibody. Upper panel demonstrating positive staining (indicated by red arrows) and lower panel indicating no positive staining in the mock (negative) control

Following exposure of cells to SARS‐CoV‐2, positivity in both N1 and N2 gene expression were observed, indicating permissiveness to infection. The mock experiments showed no evidence of viral nucleocapsid expression (Figure 6). IHC with anti‐SARS‐CoV‐2 reveals positive staining in the test sample but not in the mock control (Figure 6B).

## 4 DISCUSSION

The lack of physiological models of middle ear epithelium has hampered research efforts to understand the pathophysiology of OM and related middle ear diseases. Much work to date has relied upon animal models. For example, Mulay et al recently generated a valuable and well‐validated culture of murine middle ear epithelium.^7^ Although these have convenient husbandry and offer the ability to make genetic alterations, they are unlikely to completely reflect human disease; reasons for this include the lack of homology between animal and human genetics and also because the uniformity of laboratory animal models do not reflect the complex environments in which human diseases manifest.^8^

The other widely used tool in OM research is the human middle ear epithelial cell line (HMEEC‐1).^9^ When compared to primary cells these show marked differences both in baseline expression and stimuli‐induced expression of a range of inflammatory mediators.^10^ The HMEEC‐1 cell line also lacks important phenotypic features, such as cilia, which have known functional importance, such as mucociliary transport around the Eustachian tube orifice.

More recently, Chen et al used conditional cell reprogramming in which a Rho kinase inhibitor was used to induce a stem‐like state in epithelial cells and enable cell proliferation in human middle ear epithelium samples,^11^ however, a limitation of conditionally reprogrammed cells is that stem‐like or transiently expanded cell states do not reflect important features of differentiated cells.^12^

Overcoming these limitations would likely accelerate translational understanding of OM. Although efforts have been made to generate more physiological models of human middle ear epithelium, these have largely failed due to the small numbers of cells which are obtainable from primary middle ear mucosal biopsies.^13^

We have overcome this challenge by accessing human fetal middle ear tissue through an approved developmental biobank (HDBR; http://www.hdbr.org). The HDBR is a Wellcome Trust and MRC‐funded bioresource, which provides human fetal tissues (including ENT subsites) from 3 to 20 weeks of development for approved biomedical research in the UK and abroad in accordance with the relevant HTA and local governance Codes of Practice. Due to a larger yield of primary cells, and greater depth of sampling, we have been able to initiate and sustain traditional submerged cultures of human middle ear epithelial cells. Furthermore, we have been able to transfer these to an ALI culture system (similar to the healthy ventilated middle ear). The resultant cells are of differentiated epithelial phenotypes which recapitulate the middle ear epithelium around the Eustachian tube orifice in vivo suggesting that this may be a suitable model which, for example, could be used to study OM.

Specifically, our ALI cultures expressed typical general markers for epithelial cells (cytokeratin),^14^, ^15^ basal cells (p63), ciliated cells (FOXJ1),^16^ and (mucin) secretory subtypes (MUC5AC, MUC5B)^17^; indicating the production of a complex family of cell populations which more closely resemble the physiological environment in vivo.^18^

We also examined other markers of known importance, such as SPLUNC; an antimicrobial peptide ubiquitously expressed in the upper airways, which showed no positive staining in our cultures. It is possible that its absence is secondary to the lack of infective stimuli; indeed, this would be in keeping with the findings of Hadzhiev and colleagues who identified reduced SPLUNC expression in the middle ear mucosa of patients undergoing cochlear implantation (ie, non‐infected/inflamed mucosa) compared to those undergoing radical mastoidectomy for chronic OM with cholesteatoma.^19^

Middle ear mucosa has a key role in regulating cellular secretion and absorption of fluid and dysfunction of this process has been implicated in the pathogenesis of OM. To understand the relative roles of ion transport channels governing fluid transport in these cultures, we used the Ussing chamber with selective addition of specific channel activators and inhibitors. This revealed high ENaC function and, to a lesser extent, CFTR transporters; in keeping with previous characterization of human middle ear mucosa.^20^ Although modulation of ENaC channels with dexamethasone has been trialed in vitro^21^ treatment of OM with steroids in clinical trials has to date proved disappointing,^22^ highlighting the challenges of translating positive findings from cellular models into clinical practice.

Although OM is typically caused by a select group of otopathogens,^23^ the emerging SARS‐CoV‐2 pandemic raised the question of whether this novel coronavirus may have a role in OM and whether the middle ear could represent a clinically occult reservoir of viable virus. To date, there is retrospective evidence from postmortem examinations that SARS‐CoV‐2 can be isolated from the middle ear/mastoid^24^ and SARS‐CoV‐2 induced middle ear infection has also been inferred in clinical case reports, but these lacked prospective definitive microbiological isolation from the middle ear.^25^ Having successfully characterized our cellular model, this, therefore, represented a timely and relevant question to explore the potential utility of our system.

Direct infection of our middle ear culture demonstrated positive viral uptake as shown both by PCR and IHC. Furthermore, this positivity was observed at 24 hours post‐infection; demonstrating that middle ear epithelium is permissive to infection with SARS‐CoV‐2. The main limitation of this work is that only a single run of the infection assay was feasible due to limited access to a category 3 laboratory during the COVID‐19 pandemic. Assuming generalizability from this pilot study would suggest several important implications. First, given that initial SARS‐CoV‐2 uptake may commonly be via nasal epithelial cells; it seems probable that transmission of viral particles to the middle ear is feasible due to the migration via the Eustachian tube. Therefore, clinical signs and symptoms of OM could putatively represent middle ear infection with SARS‐CoV‐2 (COVID‐OM). Second, the presence of SARS‐CoV‐2 residing within middle ear epithelial cells also gives rise to the possibility of viral transmission in those exposed to such tissues. This may include surgical staff in the context of middle ear or mastoid surgery; particularly where the use of high‐speed drills could widely propagate virally infected cells around the operating environment.^26^ This supports the need for appropriate personal protective equipment in otological surgery. Finally, in patients with tympanic membrane perforations and chronic ear discharge who have been exposed to SARS‐CoV‐2, it seems probable that such discharge could be contaminated with infected middle ear epithelial cells, and therefore, such clinical specimens ought to be considered infectious until proven otherwise.

## 5 CONCLUSION

Herein, we demonstrate culture of human middle ear epithelial cells at an ALI, akin to the healthy ventilated middle ear. We comprehensively validate these findings and use this model to demonstrate for the first time, the permissiveness of middle ear epithelium to SARS‐CoV‐2 infection. This has important implications both for safe otological surgery and the possibility of the middle ear harboring SARS‐CoV‐2 infection.

## CONFLICT OF INTEREST

The authors declare no conflicts of interest.

## Supporting information


**Appendix S1.** Supporting Information.

**Video S1** Microscopy imaging demonstrating motile cilia

## Data Availability

All available data are in the manuscript.
