# Supplementary material for: Development of a physiological model of human middle ear epithelium
Source: Laryngoscope Investig Otolaryngol. 2021 Sep 18;6(5):1167–74. doi: 10.1002/lio2.661 (PMC8513425; doi:10.1002/lio2.661)
Supplement: Supplementary file 1 — Appendix S1. Supporting Information. [file LIO2-6-1167-s002.docx]

**Supplemental Table 1:** Antibodies and stains used in immunohistochemistry (IHC) and/or immunofluorescence (IF)

| **Protein target** | **Manufacturer** | **Species raised in** | **Dilution/**  **Concentration** | **IHC, IF or both** |
| --- | --- | --- | --- | --- |
| Cytokeratin 14/16 | St. John’s Laboratory  STJ92627 | Rabbit | 1:500 (IF)  1:200 (IHC) | Both |
| FOXJ1 | St. John’s Laboratory  STJ93116 | Mouse | 1:500 (IF)  1:200 (IHC) | Both |
| SPLUNC1 | St. John’s Laboratory  STJ95164 | Rabbit | 1:200 (IF)  1:300 (IHC) | Both |
| P63 | St. John’s Laboratory  STJ160122 | Mouse | 1:200 (IF)  1:50 (IHC) | Both |
| MUC5B | St. John’s Laboratory  STJ190985 | Rabbit | 1:50 (IF)  1:100 (IHC) | Both |
| MUC5AC | Antibodies.com  A98940 | Mouse | 1:1000 | IHC |
| MUC5AC | Abcam ab3649 | Mouse | 1:67 | IF |
| SARS-CoV-2 | Sino-biological  40589-T62 | Rabbit | 1:400 | IHC |
| Anti-mouse IgG (Alexa Fluor 594) | Thermofisher Scientific  A-11005 | Goat | 1µg/ml | IF |
| Anti-rabbit IgG (Alexa Fluor 488) | Thermofisher Scientific  A-11008 | Goat | 10µg/ml | IF |
| OmniMap anti-rabbit HRP | Ventana Medical Systems  760-149 | Goat | 30µg/ml | IHC |
| OmniMap anti-mouse HRP | Ventana Medical Systems 760-4310 | Goat | 30µg/ml | IHC |
| DAPI (4′,6-diamidino-2-phenylindole) | Sigma-Aldrich  D9542 | N/A | 1:1000 | IF |
